# Samuel Thomas Von Soemmering on the Structure of the Human Body

**Published:** 1842-10

**Authors:** 


### ART. IV.

SAMUEL THOMAS VON SOEMMERING *vom Baue des menschlichen Körpers.* —*Leipzig*, 1839-41. 8vo.

*Zweiter Band: Knochen-und Bänderlehre.* pp. 2[illegible]6.

*Dritter Band: Muskel-und Gefasslehre.* pp. 758.

*Vierter Band: Anatomie des Gehirns und Nervensystems.* pp. 752.

SAMUEL THOMAS VON SOEMMERING *on the Structure of the Human Body. Second Volume, containing the Bones and Ligaments. Third Volume: Muscles, Arteries, Veins, and Absorbents. Fourth Volume: Anatomy of Brain and Nervous System.—Leipsic*, 1839-41.

AMONGST the great anatomists of Europe few have ranked higher than Samuel Thomas von Soemmering, whether we regard the completeness, beauty, and exactness of his works, as shown in the treatise De Corporis Humani Fabricâ, and in the monographs of the Senses, or the labour and toil spent in the formation of a museum of nearly 4000 preparations. The De Corporis Humani Fabricâ has, however, ceased to exist in the library of the student, and only retains its place on the shelves of the anatomist as a work of reference. The deficiency of a general knowledge of Latin has tended to this result, as well as the publication of so many other excellent works in Europe and England on the same subject. The learned and accurate work of Hildebrandt,* and the recent manual of Bock,† have supplied its place in Germany; whilst in Bichat‡ or Cloquet§ the French student has a more pleasing source of information,

* Hildebrandt, Handbuch der Anatomie. 4 Bde. Braunschweig. 1830-32.
† Bock, C. E., Handbuch der Anatomie. 2 Bde. Leipzig, 1838.
‡ Bichat, Traité d'Anatomie descriptive. 5 Tomes. Paris, 1831.
§ Cloquet, H., Traité Descriptive. 2 Tomes, Paris, 1836.

as well as in the work of Cruveilhier,* which, with a degree of accuracy hardly ever equalled in the same subject, unites an ease and facility of expression rarely met with in professional writing. In our own country the work of Mr. Quain † unites illustration with accurate detail, whilst the dissecting-room manual of Mr. Ellis‡ stands unrivalled as a work on practical anatomy. To these may now be added the work of Cruveilhier, which the excellent translation of Dr. Madden has opened to every English student.

The volumes of Soemmering's work mentioned above form part of a new edition of the treatise De Corporis Humani Fabricâ, now publishing in Germany. This edition, when complete, will consist of nine volumes, edited (we may almost say rewritten) by the most distinguished anatomists of Germany. The second, third, fourth, and sixth volumes are published. The materials of the whole work are distributed in volumes, as follows, each subject being intrusted to the all-sufficient superintendence of the respective editors:

Vol. I. Biography of the principal Anatomists since the time of Haller, with a History of Anatomy and Physiology during the same period. By Professor Wagner, of Erlangen. (Unpublished.)

II. The Bones and Ligaments. By Professor Wagner, of Erlangen.

III. The Muscles and Vessels. By Professor Theile, of Bern.

IV. The Brain and Nervous System. By Professor Valentin, of Bern.

V. The Senses and Internal Organs. By Professor Huschke, of Jena. (Unpublished.)

VI. The Anatomy of Tissues and Chemical Composition of the Human Body. By Professor Henle, of Zurich.

VII. History of Development. By Professor Bischoff, of Heidelberg. (Unpublished.)

VIII. Pathological Anatomy. By Dr. Julius Vogel, of Munich. (Unpublished.)

IX. The Anatomy of Races and Nations. By Professor Wagner, of Erlangen. (Unpublished.)

The names of these authors are all too well known to need any comment; they are each especially fitted to complete this work of Soemmering, and add to it the discoveries and observations of later years.

In the present article it is intended to give an abstract of the contents of the volumes containing the bones, ligaments, arteries, muscles, and nervous system; the volume on general anatomy, and the part of the fourth volume devoted to the physiology of the nerves, will be noticed on another occasion.

The Museum of Soemmering is described in the first volume. This consists of 3917 preparations, and is now at Giessen. Some of these preparations were collected very early in life by Soemmering during his tour through England, Scotland, and Holland, in 1778. A very considerable part of the museum was presented by Albers, Bremser, Prochaska, Tiedemann, and others, and more especially by Behrends of Frankfort. One third part of the collection consists of pathological, the remainder of anatomical and physiological specimens. The museum contains an extensive series of the brains of animals, the original prepa-

* Cruveilhier's Anatomie Descriptive. 4 Tomes, Paris, 1834-5

† Quain's Elements of Anatomy, 4th edit. ‡ Ellis's Demonstrations of Anat. 1840.

rations for the plates of the senses, and an extensive series of embryonic preparations. Amongst the physiological preparations many are intended to illustrate the opinions of various authors, and are accompanied with references to similar cases in English and foreign works.

The account of the Bones and Ligaments is rendered more complete by notes from the other works of Soemmering, as well as from later works; much less, however, is added to this than to the succeeding volumes, as the knowledge of the skeleton and ligaments was rendered comparatively complete much before the other branches of human anatomy. The plates of Albinus and Bidloo are chiefly cited for illustrations of the bones, whilst those of Weitbrecht and Langenbeck are referred to in the description of the ligaments.

The first part of the third volume contains the Muscular System, edited by Theile, of Bern, and contains 386 pages. The plan of Soemmering is adopted, although the work itself is almost entirely rewritten. This part contains an accurate description of the anatomy and varieties of each muscle of the body, with its action and relation to the surrounding parts. In the anatomy of the muscles themselves the descriptions of Albinus, Weber, Arnold, and Santorini are especially referred to, as presenting the most accurate descriptions as well as the clearest representations. In the varieties of the muscles, the differences in origin, the number of tendons, attachments, and bellies are chiefly described: the data from which these are taken are collected in many instances from separate and detached papers in Journals and Transactions, but the most considerable part appears to be the result of the observations of the editor himself. The new muscles are divisible into two classes: those which have been described by others, but never included as a regular part of human anatomy in the common works on descriptive anatomy; and those which are first described by the editor himself. Amongst the former may be classed the reflector epiglottidis, dilatator narium posterior, musculus sacci lachrymalis, &c.; amongst the latter, the rotatores dorsi, &c. Some advantage has also been gained by ascertaining the frequency of occurrence of other muscles, as the psoas minor and levator glandulæ thyroideæ. In the relative anatomy of the muscles, the relations to the skeleton and muscular system alone are mentioned; the relative anatomy of the arteries and nerves being given in the parts especially devoted to those systems.

The part devoted to the muscles includes the fasciæ and various fibrous sheaths of the body, which are described accurately and concisely. Up to the present day the majority of anatomical works have presented a great deficiency in the descriptions of the fasciæ: when they are described accurately, the writers in many cases have aimed at originality in dividing into endless layers what is at best about as thick as a sheet of strong linen. By this mode of proceeding the student has frequently been dismayed and disheartened; and he is forced to seek the best and most accurate descriptions of the fasciæ of the body in the works of the great surgical writers. The anatomy of the head and neck, by Burns, still presents the best account of the fasciæ of this region; while the surgical writings of Sir Astley Cooper and Mr. Lawrence supply the most accurate and valuable descriptions of the fasciæ of the thigh and groin that we yet possess. The descriptions of those structures in Theile, like those by Krause, of Hanover, are plain and concise, but deficient in point and practical value, and not to be compared with those by Mr. Ellis in his recent Manual.

We shall now briefly notice the new matter which has been added to this volume: in doing so we will only mention the most important points, leaving it to the reader of the work itself to test the merit of the accurate description which abounds in every page. The first muscle revived from old works is derived from the Observationes Anatomicæ of Santorini, where an incomplete description of it exists under the name *musculus incisuræ majoris auriculæ:* it is termed by Theile

"*Dilatator conchæ.* This muscle arises from the anterior surface of the cartilaginous portion of the meatus, near the notch between the meatus and tragus; from this situation it passes downwards and outwards to the anterior surface of the tragus. *Action.* This muscle draws the tragus forwards, and thus dilates the concha." (p. 26.)

In the description of the nasal muscles some notes are inserted in reference to the opinion which Santorini, Arnold, and Tiedemann have held on this subject. The opinions expressed by them Theile considers as incorrect and imperfect. His own opinion is thus stated:

"The examination of the muscles of the nose is one of the most difficult subjects in myology. The description of the depressor, dilatator, anterior, and posterior elevator nasi is founded on numerous observations with the microscope and other means. I therefore cannot think my description of these parts superfluous. Arnold drew as compressor narium a small muscle which arose from the point of the nose and passed transversely outwards to the ala nasi, into which it was inserted. I do not know whether this is the same muscle which Santorini has represented but not named. I have never been able to find, even with the microscope, either Arnold's or Santorini's muscle. In a muscular young man I have once observed with the eye alone muscular fibres, which passed from the anterior edge of the nasal cartilaginous ala downwards and outwards in a different direction to that described by Arnold. I have never been able to find the lateralis narium of Santorini, which is represented as arising from the canine fossa of the upper jaw-bone and passing up to the ala nasi, near the anterior nasal spine. This muscle appears to me to be a part of the compressor nasi." (p. 40.)

The description given of the *dilatator nasi posterior* is the following:

"After careful separation of the fibres of the pyramidalis nasi, depressor alæ nasi, and compressor nasi, a mass of cellular tissue is observed on the inferior and posterior part of the ala nasi, in which muscular fibres may always be seen with the microscope. These fibres arise from the edge of the frontal process of the upper jaw-bone as well as from the cartilage of the alæ nasi; from this part they descend and are inserted into the posterior half of the nasal openings. *Action.* It draws the posterior half of the ala nasi backward, and dilates the nasal opening." (p. 43.)

In most anatomical works the *musculus risorius* of Santorini is described as a part of the platysma myoides; this error Theile takes pains to correct from his own observation and the original account of Santorini. This muscle is separate: it is situated on the cutaneous surface of the platysma; the fibres arise on the aponeurosis of the sterno-mastoid or platysma, and, crossing the fibres of the latter, become connected with the depressor anguli oris.

In the works of Eustachius, Santorini, Soemmering, Albinus, and Weber, a muscle has been described as *levator pharyngis internus.* This muscle is not generally described in lectures and works at the present day; but as its existence is clear from the observations of some of the best anatomists, the only excuse for the omission must be its small size; but since the compressor urethræ and muscle of Horner have attained such notoriety, it is hardly right to plead such an excuse. "The *levator pharyngis internus* arises from the lower edge of the cartilage of the Eustachian tube close to its pharyngeal extremity, and descending in the angle of the pharynx unites with the fibres of the palato-pharyngeus muscle." (p. 82.)

In the description of the *lingualis* muscle the superior portion covering the dorsum of the tongue is placed as a separate muscle from the inferior portion lying between the hyo-glossus and the genio-hyo-glossus muscles. To the former the name of lingualis longitudinalis superior, and to the latter that of lingualis longitudinalis inferior are given. The lingualis transversus of Arnold is also accurately described, but the peculiar vertical muscles of the tongue described by Gerdy* and Cruveilhier are reckoned as part of the hyo-glossus and genio-hyo-glossus muscles, and not as a separate muscle.

The *reflector epiglottidis*, previously described by Albinus and Santorini, is stated by Theile to arise from the arytenoid and inner part of the thyroid cartilage, and to be inserted into the lateral edges of the epiglottis.

In Müller's Archives, for 1839, Theile published a long account of some muscles of the back, which he named rotatores dorsi. In the present work he gives the following short description, which easily enables the student to find and recognize them.

"*Rotatores Dorsi.* On the dorsal vertebræ are found eleven small muscles on each side, which arise from the point or upper edge of each transverse process and pass to the lower edge of the arch of the vertebra above, as far as the basis of the transverse process. The first lies between the first and second dorsal vertebræ, the eleventh between the eleventh and twelfth dorsal vertebræ. The lower (with the exception of the last) are generally stronger than the superior. They are covered in their entire course by the multifidus spinæ, from which they are separated by a layer of cellular tissue. *Varieties.* The first is frequently wanting on the first and second or the eleventh, reckoning from above. A supernumerary one arises sometimes from the transverse process of the second dorsal vertebra, passes over the first dorsal vertebra and is inserted into the arch of the seventh cervical. The *action* of these muscles is to rotate the individual vertebræ on each other." (p. 164.)

Theile refers to the extensor and flexor muscles of the coccyx, described by Gunther† in his work on the Surgical Anatomy of the Muscles: these fibres arise from the lower part of the sacrum, and are inserted into the front and back part of the coccyx. They are very small, and constitute a mere rudimentary form of the large muscles moving the tails of animals.

These are the prominent novelties in the part devoted to the muscles and fasciæ; but the whole of it presents such accuracy and minuteness of detail as will well repay the reader. As a work of reference it possesses great value, and ranks with the elaborate productions of Albinus and Hildebrandt. To the learner who is acquainted with the German language this work, on account of its correctness as well as *from the descriptions* referring simply to the skeleton and other muscles, is a good book wherewith to commence the study of practical anatomy. If simply

* Gerdy, Mémoire sur la Structure de la Langue.

† Gunter, G. B., die Chirurgische Anatomie in Abbildungen. Muskellehre.—Hamburg, 1838-9. 4to.

read by itself it will appear unnecessarily minute and tedious; but if dissections be diligently performed at the same time, the minute accuracy of the descriptions cannot fail to be at once instructive and full of interest.

The second part of the third volume contains the description of the Arterial and Nervous Systems, with the absorbents, extending over 368 8vo pages. In this the editor has shown the same laborious industry which he exhibited in the part devoted to the muscles. The distribution of the vessels is described with a minute accuracy that can only have been acquired by dissection; whilst, in the enumeration of the varieties, his knowledge of the anatomical works of other countries as well as of the various memoirs and notices scattered through journals, is strikingly exhibited.

In arriving at a just arrangement of the arteries the editor appears to have experienced considerable trouble; this he attained finally by examining a number of fresh and dry dissections and establishing a standard from them, which standard he subsequently compared with a second set of dissections. In the description of the varieties all those of abortions are rejected, as belonging to morbid anatomy; a plan which does not appear to have been generally followed in previous works. No plates accompany this part of the work, but such frequent and exact references are made to the plates of Breschet, Tiedemann, Arnold, and others, that the deficiency can be easily supplied from them.

The description of the different arteries individually is well and clearly arranged. The name of the artery is placed at the head of the description with references to the best plates illustrative of it, then follow an accurate description of its relative anatomy, the measures of its length and diameter in different parts of its course, the distribution of its branches, and an enumeration of its varieties. In the part devoted to the relative anatomy, though considerable minuteness is observed, and extreme accuracy, yet the description is not so minute nor yet so advantageous to the student as in many of the English works; all the relations to various parts being mentioned with nearly the same exactness and ranked as of the same importance. Though this is just in an anatomical point of view, yet as the study of anatomy in this country is almost exclusively preparatory to the practice of the medical profession, the mode followed in the works of Cruveilhier, Harrison, Quain, and others, of setting the important surgical relations of the arteries forward in a prominent light is the most valuable and instructive to the student. In the enumeration of the various branches of the main arteries the greatest accuracy is observed; many branches which have previously only had a general description are placed as regular branches and have appropriate names given to them; this is especially the case in relation to the arteries supplying the ear, nose, and eye, in describing which the editor has made frequent reference to the works of Arnold,* Weber,† and Schlemm.‡ The varieties of each artery are given with a greater degree of minuteness and in a far more complete manner than in any previous work; and the minuteness of description is not confined to the large trunks of the body, but extends even to the minuter vessels, as those of the internal ear.

* Tabulæ Anatomicæ.—Turici. 1838 et posteà.

† Anatomischer Atlas des Menslichen Korpers.—Dusseldorf, 1836 et posteà.

‡ Arteriarum Capitis superficialium icon nova.—Berolini, 1830.

The additions made in the description of the individual vessels consist chiefly in the enumeration of new branches, in a minute description of vessels of which only a general description is usually given, and in an altered arrangement of vessels arising from the main and secondary trunks. In the new arrangement of the branches of some of the arteries the difference is chiefly confined to the smaller branches; and although the changes are calculated to add somewhat to the difficulties of studying anatomy, they seem to be justified by the attention which the editor has evidently paid to the subject.

The external and internal carotid arteries are described according to the usual arrangement, with the exception that the transversalis faciei is reckoned as one of the branches of the temporal: in most works, as well as lectures, in this country, this vessel is counted as one of the branches of the external carotid. The difference is not great nor very important, on account of the small size of this vessel. The branches supplying the internal ear are described most completely; but for the novelties in this description we must refer our anatomical readers to the original.

The arrangement of the branches of the subclavian arteries differs slightly from that usually given in English works, but not in important relations. The relative anatomy of the main trunk of the carotid artery is less complete than in some of the English works, but more complete and exact in the anatomy of the distribution of the branches, the various collateral channels of circulation about the neck and shoulder being thereby rendered very complete.

In the works of Hildebrandt, Krause, Cloquet, Cruveilhier, Ellis, and Harrison, the arteries of the spinal marrow are traced down to the lower part of the canal in front in one channel, and on the posterior part of the cord in two channels. The true and more accurate description of the circulation of these parts is given by Theile to the following effect:

"The true relation of the vessels on the anterior part of the cord is this: Two or more vessels pass through the intervertebral foramina along the anterior roots of the nerves to the anterior surface of the spinal cord as far as the anterior longitudinal fissure; here they are bent, and after having given off branches to the cord itself, unite by an ascending and descending branch with the arteries, entering in a similar manner above and below. In this manner one continuous artery is formed, receiving its blood from lateral branches. The anterior spinal arteries are less distinct than the posterior. The posterior spinal arteries cease high up. The net-shaped plexus formed by tortuous vessels, which is found on the posterior surface of the spinal cord, is formed by numerous vessels, which enter the intervertebral foramina and pass along the nerves to the back of the cord. In this situation these vessels unite with those of the opposite side as well as with those above and below. The circulation is thus carried on by currents entering laterally at certain intervals and anastomosing above and below, instead of by one descending current; each portion of the spinal marrow having thus its own peculiar set of tortuous vessels allotted to it." (p. 110.)

Into these anterior and posterior plexuses the spinal arteries are described as entering at a short distance below the skull. The description differs from that usually adopted rather in degree than in the addition of any new point; but inasmuch as it gives a more accurate account of the anatomy of an important part and one frequently referred to, it is deserving of attention. In Haller's Icones Anatomicæ we have almost the same account as that which Theile has here given.

In the arrangement of the branches of the abdominal aorta, the phrenic arteries are described as branches of the cæliac axis, and not as arising directly from the abdominal aorta ; the latter is the distribution usually taught in the English schools, and agrees with the accurate description given by Mr. Harrison, of Dublin. Haller, Bichat, and Meckel consider the phrenic arteries as branches of the cæliac axis; whilst Monro, Mayer, Boyer, Bourgery, and Hildebrandt classify them with the branches of the aorta ; all these writers, however, describe their course as variable, but that given by Hildebrandt is by far the most complete and accurate. "These arteries (phrenic) are generally two in number, but sometimes arise by a common trunk. In the latter case this trunk arises more frequently from the cæliac axis than from the aorta; in the former case, if they both arise from the same artery, it is from the cæliac axis, but the most common distribution is that where one arises from the aorta and one from the cæliac axis, the latter often being united with the coronaria ventriculi."

In the description of the arteries of the pelvis and lower extremities the chief additions consist in an accurate description of the minute arteries about the ankle and foot, to many of which especial names are given. The names are well chosen, and seem to be justly applied, not only on account of their regularity but also as rendering small vessels, which in this situation are extremely important in a surgical point of view, more known.

The description of the veins presents a complete and minute account of their normal anatomy, with a copious summary of the important varieties found to occur from time to time. In some of the branches the arrangement differs from that of other writers, but not so much as to require especial mention. In the department of the absorbents very little new matter is added to that previously described, but a complete summary from the best works is given.

The fourth volume contains the Nervous System edited by Valentin, already so favorably known by his Manual of Human Development, and his Memoirs on the Nervous System. With few exceptions this volume is an entirely new work, founded on original observations and dissections—the chief object being, in the words of the editor, "to establish a basis for the physiology and pathology of the nervous system founded on anatomy." The work is divided into three principal parts: 1st. The morphology, philosophical anatomy, and chemistry of the nervous system of vertebrate animals, and especially of man. 2d. The literature of the nervous system. 3d. The descriptive anatomy of the nervous system. The first division will be treated of elsewhere. The second division contains the anatomy of the brain and spinal cord, as well as of each individual nerve under a separate head ; the author referring particularly to scattered papers in the various journals of different countries. The manner in which this volume is written is different from that of most anatomical works. At the same time that these include the author's own observations, they present also the opinions of the best writers on the subject; but in the work before us, the text presents us with the most exact and minute descriptions without ever noticing, except very rarely, the name of any single writer on the subject; ample justice is, however, done to these in long and laborious notes. To the labour and perseverance of the author the greatest credit is due; but to the English reader, unless much time can be devoted to the study of anatomy, the work presents an uninviting aspect. It is written in hard and difficult German, arranged in some places in sentences of unusual length and complexity, and presenting a degree of minuteness in description and nomenclature remarkable even for a German work. The fifth cerebral nerve alone occupies more than one hundred octavo pages; and the synonyms of the corpora albicantia amount to twenty-five. Yet with all this minuteness and labour, the writer displays a degree of modesty and candour, which might serve as a lesson to many of far inferior industry and talent. In entering on the description of the fibres of the brain he laments the imperfection of his own description, and the want of practical knowledge as yet obtained on this subject, and looks upon its working-out as a legacy to posterity.

The work commences with the anatomy of the membranes, and description of the various surfaces, cavities, and masses of the brain, especial reference being made to the composition of each individual part, and the relations of the white and gray matter to each other. The anatomy of the brain is rendered complete, (according to our present knowledge,) by describing the various parts in their relations to each other, as shown by various sections, and by a minute description of the fibres. This part is extremely minute, and without the assistance of good plates almost unintelligible; but with the assistance of the Icones Anatomicæ of Professor Arnold, to which especial references are made, and by footnotes, it is rendered more plain and intelligible.

The account of the distribution of the nerves occupies the remaining part of the volume, amounting to about 450 pages. Although this extent may seem excessive, yet the quantity of matter is so vast, that the narrative does not appear to the attentive reader too long or capable of much condensation. The quantity of matter and information far exceeds that given by any previous writer; and at the same time that the description gives the results of the author's own dissections it overlooks none of the claims of others. In many places the previous observations of writers are arranged in an historical form with the author's comments on them, so as to give some little relief to the dry matter of detail. Each nerve is described completely, and in the following manner. The various plates of the individual nerve are named first; those of the sympathetic alone more than fill a page; the minute anatomical description of the course and branches of the nerve follows next, and then the author adds a summary of the changes in the nerve at different periods of life, and an account of the experiments which have been performed, illustrative of its physiology. Our space will only permit us to quote a few of the many observations which are of sufficient importance to render an abstract interesting.

"*Olfactory nerve.* All the three roots of the olfactory nerve are medullary and distinguishable by their white colour from the gray substance which lies near and on them; but as the gray matter covers the middle root more than the two other roots, the middle root has been supposed to be less medullary in composition than the other two. Now although the degree in which the roots are covered by gray matter varies, yet the three roots are all medullary." (p. 294.)

"*Connexion of the nasal and olfactory nerves.* I have in vain looked for a communication between the olfactory nerves and the branches of the fifth pair ramifying on the nasal mucous membrane, and have never been able to trace any communication between the first and fifth nerves in those branches, which are visible to the eye." (p. 303, *Note.*)

"*Nerves of the cornea.* In the ciliary ligament exists a considerable nervous plexus formed of the ciliary nerves; from this plexus eight or ten branches pass forwards and ramify partly in the cornea, and partly perforate the corneal margin to join the nerves of the conjunctiva." (p. 322.)

"*Connexions of the Gasserian ganglion.* The semilunar or gasserian ganglion enters into communication with other nerves in various places. 1. At the junction of the superior and internal edge, with the cavernous plexus of the sympathetic nerve surrounding the carotid artery. 2. In the same situation branches from the fourth cerebral nerve join it. 3. From the inner surface branches pass to the cavernous sinus, which, after passing through the posterior part of the body of the sphenoid bone, unite with the branches of the opposite side. 4. From the posterior and lower side of the ganglion many small fibres pass off to the cavernous and inferior petrosal sinus as well as to the neighbouring portion of dura mater." (p. 338.)

An extremely minute account is given of the relations and branches of the otic, ophthalmic and spheno-palatine ganglia. The branches of the fifth pair distributed to the lips and the branches of the portio dura distributed to the face are described seriatim, and with a degree of minuteness suited to the work as a book of reference. A clear and concise account of the distribution of the superior and inferior laryngeal nerves is given in the text, to which the opinions of Magendie, Theile,* Swan, and Müller, are added in a very complete note.

The plates of Professor Arnold are especially cited, and form almost a companion to the work. To these references are added others to the works of Bock, Langenbeck, Weber, and Scarpa; whilst the minutely accurate plates of Swan are so constantly noticed as to show on the part of the author a high esteem for our countryman, who, it must be admitted, has shown in the study of the same subject, equal industry and perseverance.

Such is a brief outline of the volumes of this great work now before us: if the succeeding parts are equally complete, this edition of Soemmering's *magnum opus* will stand first among the numerous treatises on anatomy. The brief space within which a considerable part of the work has been published justifies an expectation of its completion within a short period.

---

### ART. V.

* Theile, Dissert. de musculis nervisque laryngis.

